# Extrinsic Regulation of Optic Nerve Axon Regeneration in the Adult Central Nervous System

**DOI:** 10.3390/cells15171510

**Published:** 2026-08-22

**Authors:** Arissa Adhikary, Emily Dorairaj, Alex Arshavsky, Shanti Ramcharan, Krishna S. Kishor, Sanjoy K. Bhattacharya

**Affiliations:** 1 Department of Ophthalmology, Bascom Palmer Eye Institute, University of Miami, Miami, FL 33136, USA; arissaadhikary@ufl.edu (A.A.); emilyadorairaj@gmail.com (E.D.);; 2 Miami Integrative Metabolomics Research Center, Miami, FL 33136, USA; 3 College of Medicine, University of Florida, Gainesville, FL 32610, USA; 4 Mayo Clinic Alix School of Medicine, Phoenix, AZ 85259, USA; 5 Dr. Kiran C. Patel College of Allopathic Medicine, Nova Southeastern University, Davie, FL 33314, USA; 6 Florida Gulf Coast University, Fort Myers, FL 33965, USA

**Keywords:** optic nerve regeneration, retinal ganglion cells, glial scar, chondroitin sulfate proteoglycans, extrinsic environment, glaucoma, axonal growth, extracellular matrix, optic neuropathy, neuroinflammation

## Abstract

**Highlights:**

**What are the main findings?**

- Adult optic nerve axon regeneration after injury is not solely reliant on intrinsic factors; the extrinsic environment plays a critical role, yet it remains incompletely characterized.
- Major extrinsic barriers include alterations in the extracellular matrix, inflammatory changes, altered guidance cues, as well as vascular and metabolic limitations within the injury microenvironment.

**What are the implications of the main findings?**

- Effective optic nerve axon regeneration efforts require timing-aware, combinatorial approaches that address multiple components of the extrinsic environment in addition to intrinsic factors, rather than relying on single-target interventions.
- Patient-specific aspects such as age, disease state, and systemic comorbidities also shape environmental permissiveness and should be factored in when designing future trials.

**Abstract:**

Adult optic nerve axon regeneration has traditionally been framed as a problem of limited intrinsic growth capacity in central nervous system neurons. However, growing evidence suggests that intrinsic factors alone cannot account for regenerative failure: restrictive extrinsic environmental factors largely govern optic nerve regeneration, dictating the intrinsic capacity axons can express. In this review, we frame the extrinsic optic nerve environment as a dynamic regenerative niche, in which vascular, immune, glial, matrix, and metabolic compartments are spatially co-localized and temporally coordinated rather than acting as independent barriers. These compartments follow a shared trajectory, broadly protective in the acute phase, then inhibitory once the underlying response fails to resolve, while also actively driving one another, such as reactive astrocytes promoting the matrix remodeling that subsequently restricts axon regrowth. Consequently, the niche’s overall permissiveness for regeneration reflects the aggregate and interdependent state of these compartments rather than the action of any single barrier. This review integrates current evidence on extrinsic barriers, intervention opportunities, and disease-specific variability relevant to RGC axon regeneration after injury. These interventions must incorporate the spatial, temporal, and metabolic factors that shape the goal of functional recovery and vision restoration.

## 1. Introduction

Optic neuropathies are a group of degenerative conditions of the optic nerve that lead to irreversible vision loss [1,2]. Glaucoma, the most prevalent of these conditions, is projected to affect over 180 million people by the year 2060 [3]. The pathogenesis of glaucoma is characterized by the progressive damage and loss of retinal ganglion cells (RGCs) associated with elevated intraocular pressure (IOP) [4,5]. Although current treatments focus on preventing disease progression, they do not address existing deficits in visual field loss. There remains an unmet need for therapeutics that can promote optic nerve regeneration and reinnervation, as vision loss continues to constitute a considerable burden on patient quality of life [1,6].

Promoting axonal regrowth in the adult mammalian central nervous system (CNS) is particularly complex due to the extrinsic environmental inhibitors and the limited intrinsic regenerative capacity of neurons [7,8]. However, current perspectives on axon regeneration primarily focus on the role of intrinsic regulators, such as PTEN/mTOR pathway signaling, in dictating regeneration failure, failing to acknowledge the consequential influence of the extrinsic environment [9].

## 2. Long Distance Navigation

**Figure 1:**
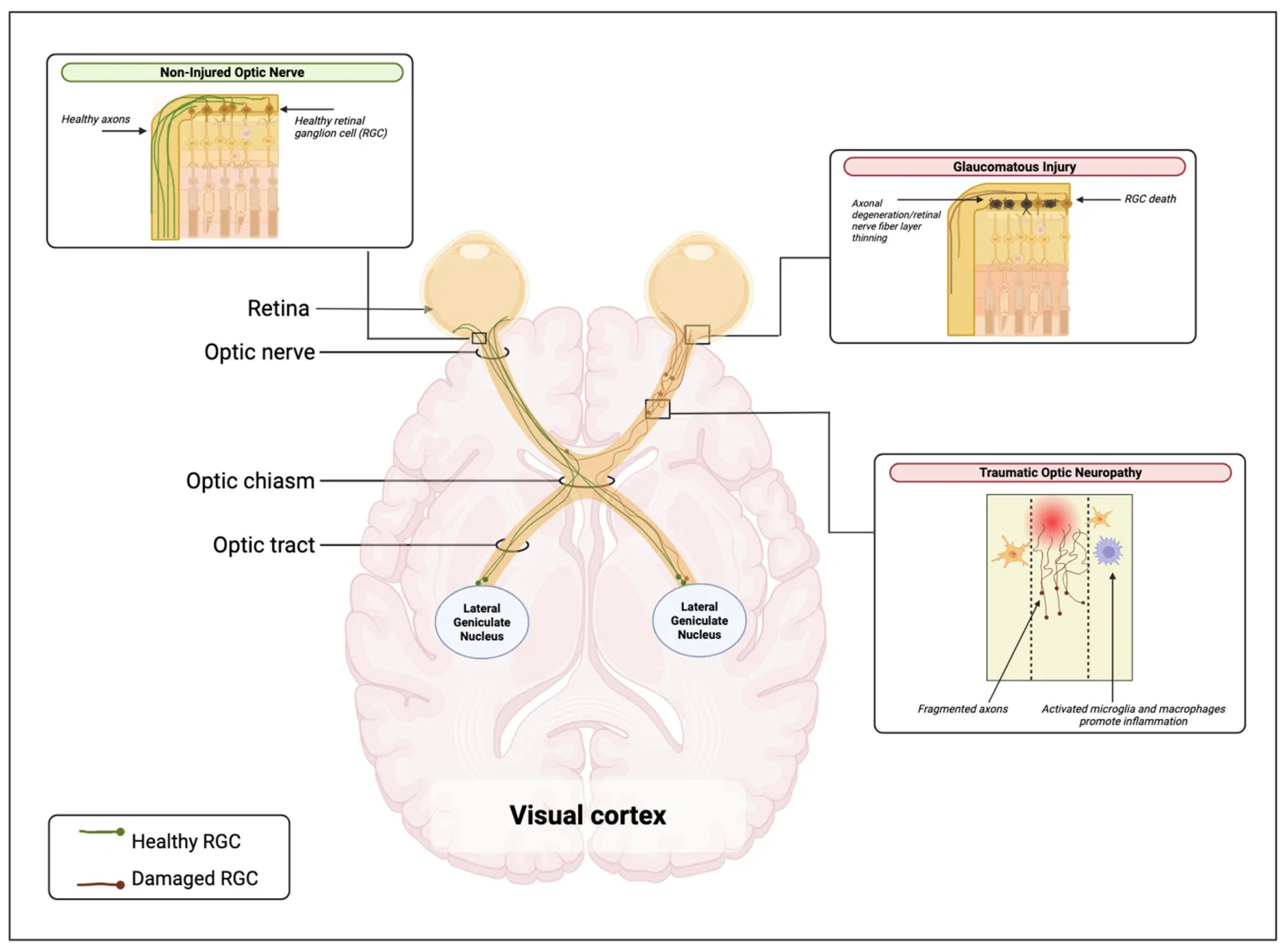
Schematic of the optic nerve visual pathway and injury response. Healthy retinal ganglion cell (RGC) axons project from the retina as the optic nerve, traveling through the optic chiasm and optic tract to the lateral geniculate nucleus. In glaucoma, progressive mechanical damage to RGCs at the optic nerve head leads to insult and death. In traumatic optic neuropathy (TON), acute injury leads to RGC damage and the formation of an injury site scar, which consists of reactive glial cells that alter the extracellular matrix and promote inflammatory signaling.

Within the context of optic nerve regeneration, we must consider the post-injury capacity of the axon to navigate the optic nerve and visual pathway to reach the lateral geniculate nucleus (LGN) (Figure 1).

The retina serves as the origin of RGCs and regulates their survival and growth signaling programs after injury [9]. For example, complex interactions in the retina may prime cells to respond to permissive cues, and changes in retinal circuit activity influence RGC growth competence and survival [9,10]. All of these factors suggest that the retinal extrinsic environment is critical for neuronal regenerative outcomes. This regenerative niche is not spatially uniform: as RGC axons extend from the retina, through the optic nerve lesion site, to their distal brain targets, they encounter different metabolic, vascular, and structural environments [11]. This period of axonal lengthening varies in compositional dominance at each anatomical checkpoint; this constitutes the bulk of challenges faced by the growing axon and is discussed in depth in the following sections. Another key site that can determine regenerative outcomes is the distal brain target. Optic nerve axon regeneration requires not only guidance during axonal extension, but also permissive signaling from active central targets, which act as distal checkpoints for reinnervation and circuit integration. Postsynaptic target neurons help regenerate RGC axons in the mature brain after injury [12]. Thus, successful regeneration requires much more than axonal survival and growth initiation. It also depends on sustained, accurate progression over long distances and correct target reinnervation, all of which is supported by growth and pathfinding factors.

## 3. Extracellular Matrix Changes

### 3.1. Injury Microenvironment

The extracellular matrix (ECM), an essential, acellular scaffold composed of interacting fibrillar proteins, glycoproteins, glycosaminoglycans, and proteoglycans, establishes the structural context for axonal growth [13]. Its composition influences cell adhesion and migration, and therefore the capability of neurons to regenerate following injury [13,14,15].

Immediately after optic nerve injury, the tissue microenvironment is altered by primary tissue damage, setting off a cascade of secondary remodeling processes [16,17]. Within the first week, CNS fibroblasts expressing Col1α1 begin to form the fibrotic scar at the lesion core, leading to an accumulation of fibroblasts and immune cells, the latter discussed in detail in Section 5 [18]. These fibroblasts have been traced to a pericyte origin, with most losing vascular association as they take on a scar-forming role [19]. Glycosaminoglycan (GAG) levels peak around seven days post-injury, coinciding with maximal astrogliosis, while uninjured controls show minimal proteoglycan staining [20].

Following the initial fibrotic response, glial scar formation begins as reactive astrocytes, oligodendrocyte precursor cells, and other immune cells infiltrate the lesion site [21]. Over the next two to three weeks, this results in a dense and compact glial scar, with further alterations in ECM composition compared to uninjured tissue [22]. The scar is made up of reactive astrocytes, microglia, and NG2+ polydendrocytes, which drive molecular ECM changes [14,23]. Notably, astrocytes upregulate the ECM glycoprotein tenascin-C post-injury, influencing glial activation and cytokine release [24,25].

As the glial and fibrotic scars mature and ECM changes persist, mechanosensitive ion channels sense the increased tissue stiffness and altered ECM composition that accompany these later stages [26,27,28]. By translating scar and ECM-associated cues into signals that suppress axonal growth, Piezo1 channels provide a direct mechanistic link between chronic microenvironmental changes and regenerative outcomes. The injured optic nerve undergoes several biomechanical changes beyond scar-associated stiffening. Substrate mechanics have been shown to directly influence axonal growth, with growing axons preferentially extending toward softer tissues [27]. Disruption of these mechanical gradients directly interferes with axonal pathfinding, suggesting that tissue biomechanics play an active role in regulating neuronal behavior [29].

Cells interpret these biomechanical cues through Piezo1 ion channels, whose activation has been shown to suppress axon regeneration through calcium-dependent signaling [30]. The role of Piezo1 also extends to glial cells, where it regulates astrocyte proliferation in the optic nerve head (ONH) [31]. Piezo1 inhibition has emerged as a promising therapeutic strategy for reducing mechanotransduction-mediated inhibition, in part due to its convenient localization on the plasma membrane, which makes it readily targetable by pharmacological agents [32].

### 3.2. Structural and Chemical Constraints

**Figure 2:**
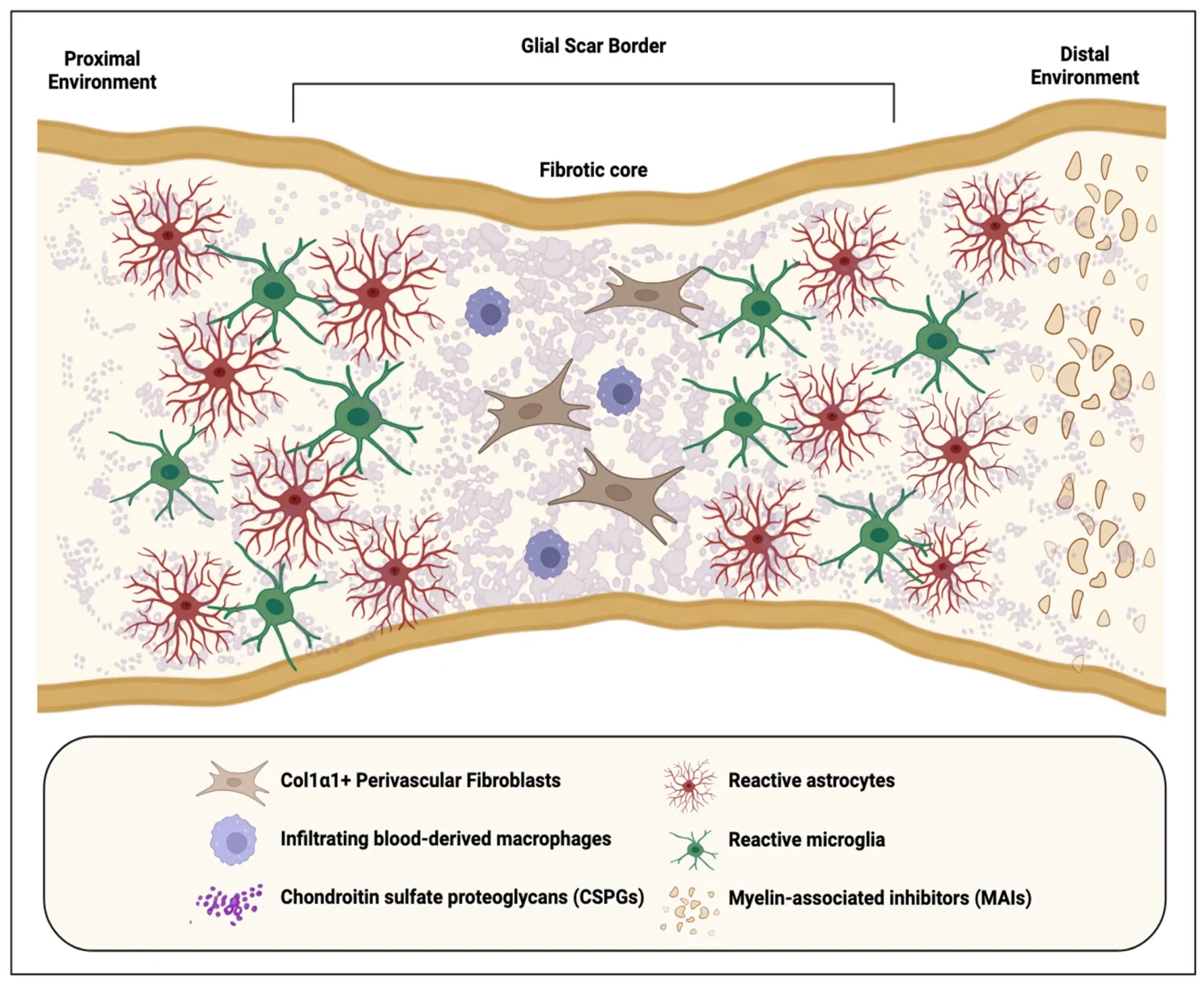
Cellular composition of the glial scar following optic nerve injury. The lesion core is occupied by fibroblasts and infiltrating macrophages. Surrounding the fibrotic core is a dense border of reactive astrocytes and microglia. Chondroitin sulfate proteoglycans are distributed throughout the scar matrix. Myelin-associated inhibitors persist in the distal environment due to incomplete debris clearance. Together, these cellular and molecular components create both structural and chemical barriers to axonal regeneration.

The glial scar initially serves a protective function by limiting the spread of the inflammatory response and subsequent cellular degeneration [33,34]. Sustained presence of unresolved scar creates a major physical barrier to axon growth (Figure 2), imposing both chemical and physical constraints [35]. The irregular geometry of the glial scar imposes a physical constraint, as its constituent astrocytes undergo restructuring into entangled filamentous processes [36]. Because RGC axons preferentially grow towards softer tissues, changes in ECM stiffness associated with the formation of the glial scar also contribute to pathfinding errors [27].

In addition to physical barriers, regenerating axons encounter spatially heterogeneous expression of chondroitin sulfate proteoglycans (CSPGs) as they traverse the lesion. In the uninjured adult human eye, CSPGs such as versican and aggrecan are present in the neural retina, choroid, and sclera [37,38]. Following injury, aggrecan and versican accumulate near the lesion site, while neurocan is more prevalent in distal regions, and brevican and phosphacan are primarily localized to the lesion core, with minimal extension into adjacent tissue, in animal models [20]. The specific molecular mechanisms by which axon outgrowth is suppressed are discussed in detail in Section 4.2, alongside myelin-associated inhibitors, receptor interactions, and growth cone modifications [39].

## 4. Chemical Cues

During development, a specialized structure found on the axon’s leading edge, the growth cone, integrates both permissive and repulsive cues to guide axons to their targets [39]. The adult optic pathway displays a fundamentally different pattern of guidance cue expression; this shift in the chemical landscape renders regeneration challenging [40,41].

### 4.1. Pro-Growth and Permissive Guidance Cues

Trophic factors are endogenously secreted molecules that regulate the survival and regenerative capability of RGCs [42]. The administration of many neurotrophic factors (NTFs), including glial cell line-derived neurotrophic factor (GDNF), brain-derived neurotrophic factor (BDNF), and ciliary neurotrophic factor (CNTF), has been investigated for its neuroprotective effects [10,43]. Trophic factors can also prime cellular responses to permissive and responsive cues. For instance, stromal cell-derived factor 1 (SDF1/CXCL12) promotes axonal growth on the permissive substrate laminin and attenuates myelin-associated inhibition [9]. Physiological electrical activity is essential for trophic factor signaling; loss of this activity impairs axonal growth even in the presence of NTFs [44]. Electrical field stimulation has been used to direct RGCs to native targets and achieve partial visual restoration [45].

NTFs exert their pro-regenerative effects by activating multiple signaling pathways. CNTF binds to a receptor complex consisting of the ciliary neurotrophic factor receptor and two transmembrane subunits, leukemia inhibitory factor receptor beta and glycoprotein 130. This interaction initiates downstream signaling through the Janus kinase/signal transducer and activator of transcription (JAK-STAT), mitogen-activated protein kinase/extracellular signal-regulated kinase (MAPK/ERK), and phosphoinositide 3-kinase/protein kinase B (PI3K/Akt) pathways [46,47,48]. The neurotrophic factors GDNF and BDNF similarly activate the MAPK/ERK and PI3K/Akt pathways via their respective receptor tyrosine kinases (RTKs) [49,50].

SDF1 signaling is mediated by activation of the C-X-C motif chemokine receptor 4, which subsequently activates the PI3K/AKT/mechanistic target of rapamycin (mTOR) pathway and elevates cyclic adenosine monophosphate (cAMP) levels. These processes enhance oncomodulin (Ocm)-mediated regeneration [9,51].

Substrates in the surrounding ECM, such as laminin, facilitate integrin-mediated adhesion and activate focal adhesion kinase (FAK), both of which are essential for regeneration. Following injury, metalloproteinases disrupt these interactions, resulting in RGC death [52]. Despite the presence of permissive substrates, ECM remodeling, persistent inhibitory debris, and additional repulsive cues collectively create an environment that restricts regeneration.

### 4.2. Guidance Cues with Dual, Repulsive and Inhibitory Functions

Post-injury, ECM remodeling leads to increases in inhibitory constituents such as CSPGs [20]. Overall, these maladaptive changes hinder the progression of the growing RGC.

CSPGs are major inhibitory cues in post-injury environments. Their biological effects are determined by specific sulfation patterns; following injury, 4-sulfated chondroitin sulfate (CS-A) motifs are upregulated compared to 6-sulfated forms [20]. The presence of 4-sulfated motifs is associated with growth cone stalling, dystrophic end-bulb formation, and the inability of axons to extend beyond the injury site. The inhibitory actions of CSPGs are further linked to the formation of dystrophic retraction bulbs that fail to regenerate and to limited collateral sprouting of spared fibers [53]. CSPGs mediate their inhibitory effects through interactions with protein tyrosine phosphatase sigma (PTPσ) receptors, leukocyte common antigen-related (LAR) receptors, Nogo receptor 1 (NgR1), and Nogo receptor 3 (NgR3) [54,55,56].

Netrin-1 functions as a guidance cue with both attractive and repulsive effects on RGC axons, depending on their progression along the visual pathway during development [57]. While expressed in adult rat RGCs, following axotomy, netrin receptors DCC and UNC-5H2 are downregulated, contrasting with observations in fish [58,59]. The absence of a functional Netrin-1 guidance system in the adult mammalian optic nerve may contribute to regeneration failure, as Netrin-1/DCC interactions and Netrin-1 scaffolding have been shown to promote induced retinal ganglion cell (iRGC) axon guidance and regeneration [60,61]. Further research should investigate approaches to upregulate Netrin-1 receptor expression in organisms with limited regenerative capacity following injury.

Myelin debris resulting from optic nerve injury contains MAIs, including MAG, OMgp, and Nogo-A. These inhibitors persist in the injured CNS and prevent the growth cone from advancing [62,63]. MAG, OMgp, and Nogo-A exert their effects through a limited set of shared receptors, most notably NgR1 and paired immunoglobulin-like receptor B. The shared binding of CSPGs and MAIs to the NgR receptor family indicates overlapping inhibitory mechanisms [56].

Additional repulsive cue classes further contribute to the inhibitory environment. Semaphorin 5A, which is expressed exclusively by oligodendrocytes and their precursors, inhibits axonal growth and induces growth cone collapse [64]. Semaphorin 3A has been shown to activate M1 microglia, resulting in RGC death [65]. This molecule inhibits axon growth via the neuropilin-1 and plexin A1-4 receptor complex [66]. Ephrins, which are membrane-bound repulsive cues, interact with RTKs. Notably, knockout of ephrin type-A receptor 4, an RTK that binds ephrinA ligands, substantially increases axon regeneration and promotes migration of glial fibrillary acidic protein-positive astrocytes from the perilesional area [67]. Slit homolog 1 is re-expressed in the adult optic chiasm 10 days after optic nerve injury and primarily prevents the growth of RGCs expressing roundabout guidance receptor 2 outside the optic chiasm. However, this expression does not persist at six weeks post-injury, suggesting a failure of sustained growth, potentially due to the absence of this cue [41].

Despite the potential for long-distance axon regeneration, persistent inhibitory signals and debris in the adult visual system can misdirect regenerating axons and hinder circuit repair [68]. Modulating such cues may help restore functional neural circuits.

**Figure 3:**
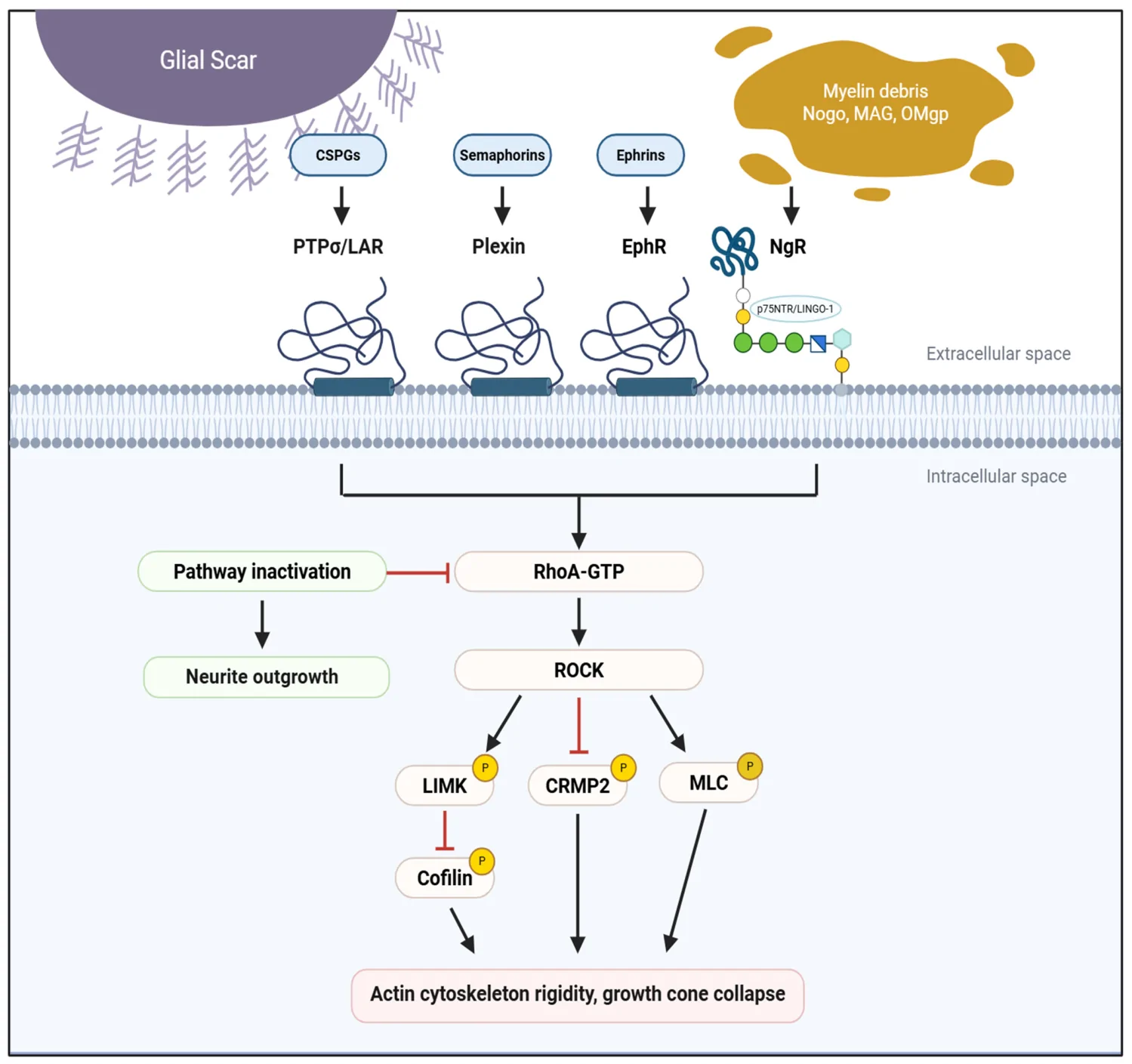
Convergent inhibitory signaling through the RhoA/ROCK pathway. Multiple classes of extracellular inhibitory molecules, such as chondroitin sulfate proteoglycans (via protein tyrosine phosphatase sigma/leukocyte common antigen-related receptors), semaphorins (via plexin receptors), ephrins (via Ephrin receptors), and myelin-associated inhibitors including Nogo-A, myelin-associated glycoprotein, and oligodendrocyte myelin glycoprotein (via nogo receptors), converge intracellularly on RhoA-GTP activation. GTP-bound RhoA activates ROCK, which phosphorylates downstream effectors including LIM kinase, collapsin response mediator protein 2, and myosin light chain. Pathway inactivation, achieved through RhoA or ROCK inhibition, permits neurite outgrowth.

The convergence of these signals onto a single intracellular pathway strongly supports their complementary roles within the axon microenvironment, rather than independent functions. Many inhibitory signaling molecules, including certain CSPGs, MAIs, semaphorins, and ephrins, exert their effects by modulating the (RhoA/ROCK) pathway, as seen in Figure 3 [66,69,70,71]. In this sequence, GTP-bound RhoA activates ROCK, which then mediates actin stabilization and cytoskeletal remodeling through downstream effectors such as LIM domain kinase, cofilin, and collapsin response mediator protein 2 (CRMP2) [72,73,74]. Ultimately, these effects lead to the inhibition of neurite growth and growth cone collapse. Therefore, targeting the RhoA/ROCK pathway may provide a means to simultaneously modulate the effects of multiple growth-inhibitory cues (Table 1) [10,40].

## 5. Inflammatory Responses

Inflammatory responses following optic nerve injury exert a dynamic and time-dependent influence on axon regeneration. Instead of being exclusively supportive or inhibitory, inflammation progressively alters the extrinsic environment, transitioning it from a transiently growth-permissive state to a growth-restrictive one [132].

### 5.1. Acute Inflammatory

It has been widely characterized that optic nerve injury initiates axonal severance followed by Wallerian degeneration [133]. During the early post-injury period, myeloid-derived macrophages infiltrate the injury site, facilitating debris clearance and releasing pro-regenerative factors such as Ocm [134,135]. At this injury phase, RGCs adopt a growth-competent state that enables axon extension in response to inflammatory cues [136]. Chemokines, including C-C motif chemokine ligand 2 (CCL2) and C-X-C motif chemokine ligand 5 (CXCL5), promote macrophage infiltration and contribute to transient pro-regenerative signaling [132]. Complement signaling is rapidly upregulated, involving complement components 1q and 3, primarily at the optic nerve [135]. Vascular changes occur rapidly after injury. In TON models, nine hours after crush injury, leukocyte rolling and adhesion are observed in veins near the optic nerve head, preceding RGC dysfunction [137]. At the same time, resident microglia are rapidly activated in response to axonal degeneration and myelin debris. However, their phagocytic capacity remains insufficient for complete debris clearance [134].

### 5.2. Transition to Chronic State

With incomplete debris clearance, MAIs, sulfatide lipids, and other growth-restrictive components accumulate over time [138]. Sustained inflammatory signaling, including complement, interleukin-1α, and tumor necrosis factor-α pathways, induces astrocytes to adopt neurotoxic reactive states [139], subsequently driving peak CSPG deposition as discussed in Section 3.1. While acute complement activation facilitates debris clearance and regeneration, prolonged or dysregulated activation can trigger inflammasomes such as NOD-like receptor protein 3 (NLRP3), resulting in a progressively inhibitory environment [134,140]. Chronic immune activation compromises the blood-retinal barrier, leading to dysfunction of the retinal neurovascular unit. Fourteen days after crush injury, levels of potassium channel Kir4.1 and tight junction proteins claudin-1 and claudin-5 decrease, while aquaporin-4 (AQP4) increases, reflecting broader disruption of the blood-retinal barrier following optic nerve injury [141].

Inflammatory responses evolve over the course of injury, and the effects depend on timing and structure. Following day 1, monocyte-derived macrophages arrive at the optic nerve once activated microglia have cleared RGC debris [142]. By day 3, astrocytes degenerate at the optic nerve, creating a regeneration-prohibitive environment through the formation of the glial scar [142]. The glial scar forms a pathway for resident microglia and infiltrating macrophages; the increasing number of macrophages can overcome the glial scar to create clearance of myelin debris and regeneration of axons across the glial scar [143]. The CNS microglia and macrophages transition from a neurotoxic to neuroprotective phenotype [143]. This emphasizes that immune responses do not follow a straightforward transition; rather, contingent upon spatial and temporal factors.

Overall, the transition from acute to chronic neuroinflammation establishes a persistently non-permissive environment that extends beyond the initial injury [17,144,145]. Prolonged debris accumulation and sustained immune activation expose RGC axons to adverse conditions, rendering regenerative outcomes dependent on the timing, magnitude, and resolution of inflammation [146]. Alongside ECM remodeling and guidance dysregulation, these barriers reinforce growth-inhibition that persists far after injury (Table 2) [16,147].

## 6. Metabolic Constraints

The microenvironment associated with optic nerve injury imposes significant energy constraints on injured neurons, further worsening the structural and chemical alterations that impede regeneration. Metabolic stress largely results from the vascular and immune changes discussed in Section 5 and Section 7.

### 6.1. Mitochondrial Dysfunction Due to Injury

Optic nerve injury disrupts axoplasmic transport and decreases the expression of proteins essential for mitochondrial homeostasis, such as Vps35, resulting in mitochondrial dysfunction [152]. This impairment diminishes the energetic capacity of injured axons and modifies growth cone responses to environmental cues [153]. Oligodendrocytes directly support axon metabolism by sensing axonal activity through Kir4.1 channels and supplying lactate in response. Loss of this signaling has been associated with axon damage in other CNS injuries [154]. Furthermore, reduced availability of metabolic substrates during periods of elevated energy demand compromises the energy supply necessary for axonal growth and guidance. For instance, mitochondrial triglyceride depletion contributes to secondary axonal degeneration and diminished regenerative capacity [155]. These findings suggest that the injured microenvironment lacks the metabolic infrastructure required to support long-distance axonal regeneration.

### 6.2. Metabolic Adaptation and Lipidomics

Experimental models demonstrate that as mitochondrial function deteriorates, injured axons increasingly depend on glycolysis for rapid adenosine triphosphate (ATP) production. Although this metabolic adaptation provides limited support, it does not sustain robust regeneration when mitochondrial metabolism is impaired [156]. Vascular hypoperfusion and chronic immune activation add to this burden. Both promote HIF-1α accumulation and shift cells toward less efficient glycolytic metabolism [157,158]. Beyond mitochondrial alterations, lipidomic analyses following indirect optic neuropathy reveal a time-dependent accumulation of sphingomyelin and ceramide species. These changes are associated with altered membrane properties, activation of apoptotic pathways, and the development of a non-permissive environment [159]. Collectively, the effects of mechanical resistance and metabolic stress further reduce ATP bioavailability and limit growth cone progression, hence reinforcing an environment that inhibits regeneration.

## 7. Specific Disease States

**Figure 4:**
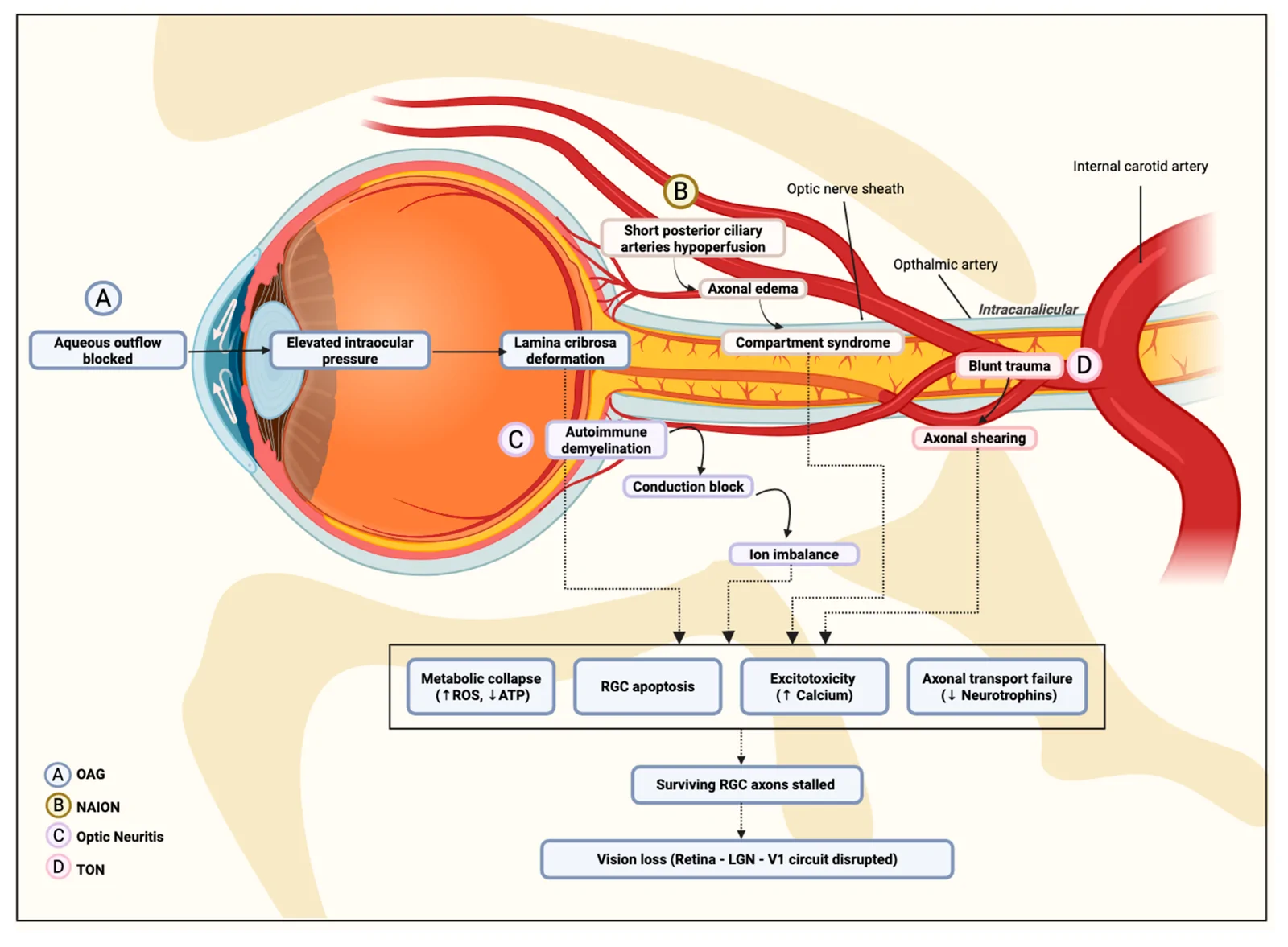
Disease-specific upstream mechanisms converging on a shared optic nerve injury cascade. Four optic neuropathy contexts, open-angle glaucoma, non-arteritic anterior ischemic optic neuropathy, optic neuritis, and traumatic optic neuropathy, are shown with their early pathological mechanisms. Despite distinct upstream etiologies, all four pathways converge on a common injury cascade involving retinal ganglion cell (RGC) death signaling, metabolic collapse, reactive gliosis with extracellular matrix remodeling, and axonal transport failure. Surviving RGC axons are then subject to compounding extrinsic and intrinsic blocks, ultimately resulting in vision loss through disruption of the retina, lateral geniculate nucleus, and V1 circuit.

The extrinsic environment of the optic nerve varies according to disease mechanisms underlying optic neuropathies, as well as age-related factors and comorbidities, as seen in Figure 4 [160]. Optic nerve crush (ONC) models are commonly used for optic nerve injury, as the applied crush injury causes gradual RGC death [161,162]. Nevertheless, ONC models do not fully recapitulate the extrinsic environments characteristic of chronic or mechanically mediated optic neuropathies, including glaucoma and traumatic optic neuropathy (TON).

### 7.1. Disease-Specific Pathological Mechanisms

Although these disease states originate from distinct upstream causes, they converge within a common downstream environment. This convergence underscores that the interconnected roles of vascular, glial, immune, and ECM components are not limited to a single injury mechanism; instead, these roles are complementary and highly interdependent. For instance, in ONC models, microglial activation primarily acts as a bystander during RGC damage [163]. In contrast, early-stage glaucoma is characterized by increased microglial proliferation and activation after IOP elevation, indicating a causal role for neuroinflammation in glaucoma pathogenesis [164,165,166,167]. Activated microglia promote the transformation of astrocytes into a neurotoxic A1 phenotype, a process known as astrogliosis [168,169]. These A1 astrocytes upregulate complement component C3, which mediates synaptic destruction and leads to the death of oligodendrocytes and RGCs [170,171]. Additionally, reactive astrocytes contribute to ECM remodeling in the lamina cribosa in response to IOP elevation [172]. Experimental studies using ONC or transection demonstrate that these fibrotic changes are specific to IOP-induced mechanical stress rather than being a consequence of RGC injury alone [173,174]. In TON, mechanical injury initiates the pathological process, while secondary vascular compromise modulates the severity of damage and accounts for variability in visual recovery [175]. In contrast, non-arteritic anterior ischemic optic neuropathy (NAION) arises when hypoperfusion or atheromatous disease interacts with anatomical crowding of the ONH [175]. These conditions illustrate how vascular dynamics additionally influence the permissiveness of the extrinsic environment.

Optic neuritis (ON) is a frequent, acute inflammatory injury to the optic nerve found in adults and children [176]. The inflammatory disorders associated with ON in the CNS include multiple sclerosis (MS), aquaporin-4 (AQP4)-IgG-, and myelin oligodendrocyte glycoprotein (MOG)-IgG-associated disease [177]. In clinical settings, MS-associated ON is unilateral, moderate, painful vision loss with an afferent pupillary defect and normal fundus examination; if bilateral vision loss and pain are present, alternative optic neuropathies are to be considered instead [176]. Visual recovery begins 2–3 weeks post-injury, improves rapidly through the first month, then slows over the following months [178].

Acute ON results in inflammatory swelling of the RNFL of the affected eye that resolves within 3 months, followed by RNFL thinning that is most prominent after 6 months and lasts up to 1 year [178]. Swelling during acute ON may mask early RNFL thinning; Spectral Domain Optical Coherence Tomography (SD-OCT) is used in these cases to track the timing of axon loss more precisely [178]. Inflammatory responses initially create swelling that can affect when structural changes occur and are evaluated. Therapeutic strategies aim to suppress inflammation during the acute phases, so stages of injury must be determined to increase the ability for regeneration.

### 7.2. Experimental Limitations

While ONC models give useful insights into extrinsic factors contributing to optic nerve degeneration, differences in disease onset and progression should be considered when translating these findings to clinical optic neuropathies. The absence of spontaneous-type animal models for primary open-angle glaucoma (POAG) remains a significant limitation; the development of such models may address these translational challenges in the future [179]. Furthermore, ONC models are limited in their ability to model TON, as they do not replicate the shearing injuries typically observed in patients with blunt force trauma [180].

Overall, heterogeneity in the extrinsic environment strongly affects both degenerative processes and regenerative potential. This points to the necessity of disease-specific interpretation of experimental models to accurately capture context-dependent effects.

## 8. Modifier States That Precondition the Extrinsic Environment

Age and systemic comorbidities add to extrinsic barriers by affecting the timing and extent of the transition from acute to chronic phases. This, in turn, influences whether the optic nerve niche becomes more permissive or inhibitory after injury.

### 8.1. Age

Deletion of the phosphatase and tensin homolog (PTEN) gene, an axonal growth inhibitor, upregulates the mTOR pathway and promotes axonal growth [181]. Despite this intrinsic activation, in the spinal cords of older individuals, many axons still do not extend beyond the injury site, where astroglia and inflammatory markers are upregulated [182]. These observations suggest that age-related declines in regeneration are due to a shift in the extrinsic environment of the CNS rather than intrinsic failure.

Even before injury, age-dependent changes promote ECM remodeling, RGC loss, and compensatory glial activation in the optic nerve [183]. Over time, ECM components increase in thickness and stiffness, which increases disease susceptibility [184]. In the context of optic nerve regeneration, it may pose a challenge to RGC outgrowth [29].

Studies in highly regenerative species indicate that aging alters immune responses following optic nerve injury, leading to dysregulated inflammation and diminished pro-regenerative signaling. For instance, experimental findings in zebrafish and killifish demonstrate that aging increases abnormal glial cell activation, thereby limiting regenerative potential [185,186].

These findings show the growing significance of age-related changes in the extrinsic environment. Further research is needed to elucidate the underlying mechanisms, particularly in the context of optic nerve injury in organisms with limited regenerative capacity. In clinical practice, age should be considered as a factor contributing to patient-specific variability in responses to optic nerve injury.

### 8.2. Comorbidities

Obstructive sleep apnea (OSA), blood pressure dysregulation, and diabetes mellitus (DM) are systemic disorders that adversely alter the extrinsic environment of the ONH by inducing vascular dysfunction and oxidative damage. These cellular and metabolic alterations increase the ONH’s vulnerability to injury.

In OSA, recurrent apneas result in intermittent hypoxia. This promotes oxidative stress, inflammation, and endothelial alterations in ONH vessels [187,188]. Hypoxia disrupts the balance between nitric oxide and endothelin-1. This imbalance leads to vascular dysregulation and elevated intracranial pressure, especially during nocturnal systemic hypotension [189]. Intermittent hypoxia also activates the sympathetic nervous system. This increases systemic blood pressure through vasoconstriction [190]. These cyclical changes impair blood flow to the ONH and create an unfavorable environment for optic nerve regeneration.

These OSA-related vascular mechanisms demonstrate how blood pressure regulation shapes the optic nerve’s microenvironment in glaucoma. Systemic blood pressure adjusts the blood supply to the ONH by controlling ocular perfusion pressure (OPP) [191]. Chronic hypertension stresses vessels and thickens vessel walls, which encourages plaque formation and ischemic injury [191,192]. These changes reduce blood flow and nutrient delivery, leading to axonal stress and glaucomatous damage.

In addition to hemodynamic factors, metabolic disorders such as DM alter the extrinsic environment of the ONH by promoting inflammation and glial cell dysfunction [193]. Experimental models of diabetes demonstrate changes in astrocyte activity, including increased glial fibrillary acidic protein expression, indicating disruption of normal astrocyte function [194]. These changes are characteristic of astrogliosis and may exacerbate cellular and molecular changes in neural injury by inducing pro-inflammatory and apoptotic microglial mediators [195,196]. Thus, DM disrupts glial cell homeostasis, creating a more vulnerable extrinsic environment for RGCs and promoting the progression of optic neuropathies.

## 9. Molecular Targets and Modulation Agents

Current therapies for glaucoma focus on lowering IOP; however, elevated IOP is not present in many patients who display glaucomatous progression, indicating that mechanisms independent of IOP may play a role in the disease [197]. This emphasizes the necessity of alternative mechanisms of disease control [198]. Building on this perspective, in the context of TON, targeted agents remain limited and lack evidence-based support, highlighting a gap in treatment for optic nerve injuries [199,200].

Potential treatment for optic neuropathies should also aim in part to optimize extrinsic architecture to promote sustained regeneration. Intervention into the extrinsic environment may be best understood not as directly inducing regeneration, but as establishing the conditional environment under which regeneration becomes possible.

Here, we outline potential molecular targets and identify modulators that alter the extrinsic environment and its downstream effects on neurons, supporting axonal survival and regrowth (Table 3). As many molecules that contribute to axon regeneration are similarly expressed in optic nerve and spinal cord injuries, we can infer that there may be shared mechanisms across the CNS for regeneration and failure. Although the following subsections are organized by mechanism for clarity, single-node interventions often have limited clinical impact. The optic nerve ecosystem is highly interconnected and cannot be addressed in isolation, as further discussed in Section 10.

### 9.1. Extracellular Components

Following CNS injury, the accumulation of CSPGs in the glial scar forms a major barrier to axonal regrowth. CSPG GAG chains bind neuronal receptors and disrupt growth cone progression. Carbohydrate sulfotransferase-11 (CHST-11) adds sulfate groups at the 4-position of chondroitin sulfate chains, thereby regulating 4-sulfation patterns and shaping the inhibitory properties of CSPGs in the post-injury ECM. Inhibiting CHST-11 during early glial scar formation may therefore reduce the production of highly inhibitory 4-sulfated CSPGs within the optic nerve injury environment [201].

Specifically targeting GAG chains after scar formation is another promising approach. Enzymes such as chondroitinase ABC (ChABC) and hyaluronidase, which degrade GAG side chains, have been shown to promote axonal extension in multiple experimental models [202,203,204]. Arylsulfatase B (ARSB), which specifically removes 4-sulfated residues (4S) from the non-reducing ends of GAG chains, can neutralize inhibitory signals in a more targeted way while preserving the ECM structure [205]. These findings suggest that modulating rather than eliminating components of the extracellular environment may be sufficient to support growth after injury. Taken together, these findings present a potential role for CSPGs as regulators of growth cone behavior, which can be modulated to promote optic nerve regeneration.

ECM-based tissue-engineering therapies are also promising candidates for promoting long-term optic nerve regeneration through their multifactorial benefits [206,207]. Naturally derived ECM bioscaffolds are produced through decellularizing mammal-derived tissues. This process retains growth-promoting molecules such as collagens, glycosaminoglycans, laminins, and growth factors, while also eliminating growth-inhibitory molecules such as MAGs and CSPGs [206,207,208,209].

Synthetic optic nerve scaffolds, such as those made from poly (ε-caprolactone) (PCL) and poly-gamma-benzyl-L-glutamate (PBG), are also being developed due to their use in improving cell viability and growth [206,210,211,212]. However, these treatments are far from clinical translation, as ongoing research has only just begun to evaluate tissue engineering techniques in animal CNS regeneration [209].

Beyond providing structural support, emerging scaffolding strategies are integrating electrical stimulation to create a more permissive regenerative environment. Electrical stimulation has shown several neuroprotective effects, particularly improving photoreceptor function and RGC survival following transection, as well as preserving retinal function following injury [213,214,215,216]. These neuroprotective effects are thought to arise from increased production of neurotrophic factors, including BDNF and insulin-like growth factor 1 (IGF-1), by Müller cells [213,214]. More recently, electrical stimulation has emerged as an effective strategy for promoting long-distance axon regeneration, with applied electrical fields partially restoring vision following ONC [45]. Electrically conductive biomaterials, including injectable hydrogels, have also been developed; for example, poly(3,4-ethylenedioxythiophene) (PEDOT) hydrogel enhances RGC survival by reducing apoptosis, highlighting the therapeutic potential of combining electrical stimulation with scaffold-based therapies [217].

### 9.2. Inhibitory Signaling

Neutralizing neuronal responses to inhibitory signals offers an alternative strategy for promoting axonal regrowth, as opposed to directly modifying the extrinsic environment. 

At the receptor level, PTPσ and other members of the LAR subfamily bind CSPGs and have thus been studied as potential targets for intervention. While PTPσ knockout mice display diminished CSPG-mediated inhibition in the CNS, misdirection errors in regenerating axons have been observed in sciatic nerve injury models, raising concerns regarding complete receptor knockout [53,218]. This finding highlights the risks of single-target interventions. Controlling one signal without accounting for environmental barriers may disrupt axonal balance, emphasizing the need for multimodal strategies that consider all relevant factors. In the spinal cord, inhibition of PTPσ and LAR using ILP/ISP decreases the number of activated M1 microglia/macrophages, while simultaneously promoting the activation of M2 microglia/macrophages and T regulatory cells [219]. Another potential receptor target is NgR1, which binds both CSPGs and MAIs. The administration of intravitreal NgR1(310)-Fc, an NgR1-blocking decoy, has been shown to promote regeneration after ONC and to demonstrate neuroprotective effects in a microbead glaucoma model [220].

Downstream, CSPGs, MAIs, and other repulsive guidance cues activate RhoA, a member of the Rho family of small GTPases, which mediates growth cone collapse and cytoskeletal remodeling [221]. Inactivation of RhoA using C3 transferase, Y-27632, or Y-39983 reduces inhibitory signaling and permits neurite outgrowth on inhibitory substrates, affecting both compartments simultaneously [73,222,223]. Because many inhibitory molecules regulate axon regrowth through RhoA-dependent pathways, targeting this signaling cascade may effectively counteract extrinsic inhibitory influences.

### 9.3. Trophic Factors 

In addition to targeting growth-inhibitory pathways, the activation of growth-permissive pathways represents an equally essential aspect of the regenerative process.

NTFs such as CNTF, BDNF, and GDNF have shown promise as neuroprotective agents, especially when administered in combination [43,224,225,226]. Recently, stem cell transplantation has emerged as a potential therapeutic strategy for glaucoma, potentially replicating the effects of NTF treatment alongside other environmental modifications. Beyond secreting NTFs, mesenchymal stem cells are believed to play roles in immunoregulation and in preventing RGC apoptosis. However, optimal administration, as well as the safety and efficacy of these approaches, remain to be established [227].

Infiltrating myeloid cells express the axogenic protein Ocm, which is enhanced by SDF1, mediated by intracellular cAMP elevation and phosphatidylinositol-4,5-bisphosphate 3-kinase (PI3K). This process partially restores axonal regeneration. When SDF1 is combined with inflammation and PTEN deletion, SDF1 enables a larger population of RGCs to traverse along the optic nerve [51].

### 9.4. Immune and Glial Responses

Immune and glial responses play critical roles in determining axonal regeneration after injury. Inflammation demonstrates dual functions, as specific activation states may either facilitate or inhibit axonal growth. Contemporary strategies aim to enhance adaptive, acute immune responses while minimizing chronic inflammatory damage.

After lens injury (LI), infiltration of neutrophils and macrophages enhances RGC survival and axonal outgrowth through the secretion of Ocm [132]. In contrast, intravitreal administration of the toll-like receptor 2 (TLR-2) ligand zymosan, a yeast cell wall carbohydrate, stimulates macrophages without causing injury [228]. The regenerative effects of LI or zymosan are mediated by inflammatory chemokines, including CCL2 and CXCL5. Blocking these receptors impairs axonal growth, while stimulation with the TLR2 agonist Pam3Cys or the Dectin-1 agonist β-glucan can reproduce these regenerative outcomes [132].

Microglia–astrocyte signaling contributes to neurotoxicity; thus, modulating this interaction has emerged as a promising strategy to enhance neuronal survival and axonal regeneration. Inhibition of Sema3a-mediated microglial activation by blocking CRMP2 phosphorylation reduces microglial activation and improves RGC survival [229]. Glucagon-like peptide-1 (GLP-1) agonists also attenuate this signaling and have been investigated for their ability to shift inflammatory responses toward a neuroprotective phenotype. Recent evidence indicates that GLP-1 agonists may lower the risk of POAG development [230]. Astrocyte activation increases C3 expression; however, treatment with NLY01, a GLP-1 agonist, effectively inhibits C3 production by suppressing microglial inflammation and preventing astrocytes from adopting a neurotoxic state [139,231,232]. Collectively, these mechanisms and therapeutic interventions represent potential strategies to promote RGC survival in glaucoma.

### 9.5. Toward Combinatorial, Time-Sensitive Interventions

The application of the therapies discussed above remains largely theoretical, as many have only been validated in animal or spinal cord regeneration models, which may not be directly translatable to human optic nerve regeneration. Successful future approaches will most likely be combinatorial, addressing multiple aspects of the extrinsic regenerative environment in tandem and at an optimal window of intervention. However, promising large-scale regenerative therapies such as stem cells still face substantial barriers before they are suitable for clinical use.

The findings presented in this review indicate that optic nerve injuries should be viewed as a continuous, evolving dynamic environment rather than a cascade of independent barriers. Early ionic dysregulation disrupts signaling and destabilizes the microtubule cytoskeleton [233,234]. Wallerian degeneration initiates swelling and myelin breakage [142]. Mitochondrial dysfunction increases reactive oxygen species (ROS) production and decreases ATP synthesis, which limits the amount of energy and compromises the ability of RGCs to regenerate [235]. Importantly, these processes have overlapping timelines and can stimulate each other, shifting the environment away from a regenerative state.

**Table 3:** Extrinsic molecular targets.

Extrinsic Molecular Targets	Intervention	Repair Mechanism
**Extracellular matrix**	ChABC [203,204]	Cleaves CS-GAG sidechains, freeing CSPG from the ECM
	Hyaluronidase [202,236]	Digests the GAG hyaluronan, reducing CSPG interactions with the ECM
	ARSB [205]	Removes 4-sulfated groups from non-reducing ends of CSPG GAG chains
	CHST-11 inhibition [201]	Regulates CHST-11-mediated 4-sulfation of CSPGs
	ECM Bio-scaffolding [208,209]	Supplements pro-growth molecules and reduces inhibitory molecules such as CSPG and MAG
	ECM Synthetic scaffolding [210,211,212]	Improves cell survival and guides adaptive growth
**Inhibitory Signaling**	PTPσ knockout [53,218]	Achieves genetic knockdown of the CSPG receptor PTPσ, thereby reducing CSPG-mediated inhibition
	ISP/ILP peptides [219]	Bind and inhibit PTPσ and LAR receptors intracellularly, reducing CSPG inhibitory signaling
	NgR1(310)-Fc [220]	Soluble decoy protein that sequesters NgR1 ligands, blocking inhibitory myelin signaling
	Y-27632 [237]	Inhibits Rho-associated protein kinase (ROCK), blocking RhoA/ROCK signaling
	Y-39983 [223,238]	More potent ROCK inhibitor derived from Y-27632
	C3 transferase [73]	Inactivates Rho via ADP-ribosylation to inhibit RhoA/ROCK signaling
**Trophic Factors**	CNTF [239]	Primarily binds to CNTFRα, recruiting signal transducing receptors LIFRβ and glycoprotein 130 to form a tripartite complex which activates JAK/STAT, PI3k/Akt, and MAPK/ERK pathways
	BDNF [240]	Signals through TrkB receptors to promote axon growth through MAPK/ERK and PI3k/Akt pathways
	GDNF [239]	GDNF primarily forms a GDNF-GFRα1 complex which activates receptor tyrosine kinase (RET) to activate MAPK/ERK and PI3K/Akt pathways
	SDF1/CXCL12 [241,242,243]	Activates the CXCR4 receptor, leading to downstream PI3K/Akt, MAPK/ERK, and JAK/STAT pathway activation.
	Mesenchymal stem cells [227]	Release trophic factors and anti-apoptotic molecules, and mediate several aspects of immune response, promoting RGC survival
**Immune Signaling**	Lens injury [228]	Induces inflammation and the infiltration of oncomodulin-secreting macrophages/neutrophils
	Zymosan [228]	Mimics the effects of lens injury via stimulation of TLR2 and Dectin-1 receptors
	Glucagon-like peptide-1 agonists [139,231,232]	Activates GLP-1 receptor signaling, reducing microglial transformation of astrocytes into a neurotoxic phenotype
	CRMP2 modulation [229]	Regulates the semaphorin-mediated phosphorylation state of CRMP2, sustaining its dephosphorylated form, which promotes microtubule formation

## 10. Synthesis and Future Directions

### 10.1. Integrated Model

**Figure 5:**
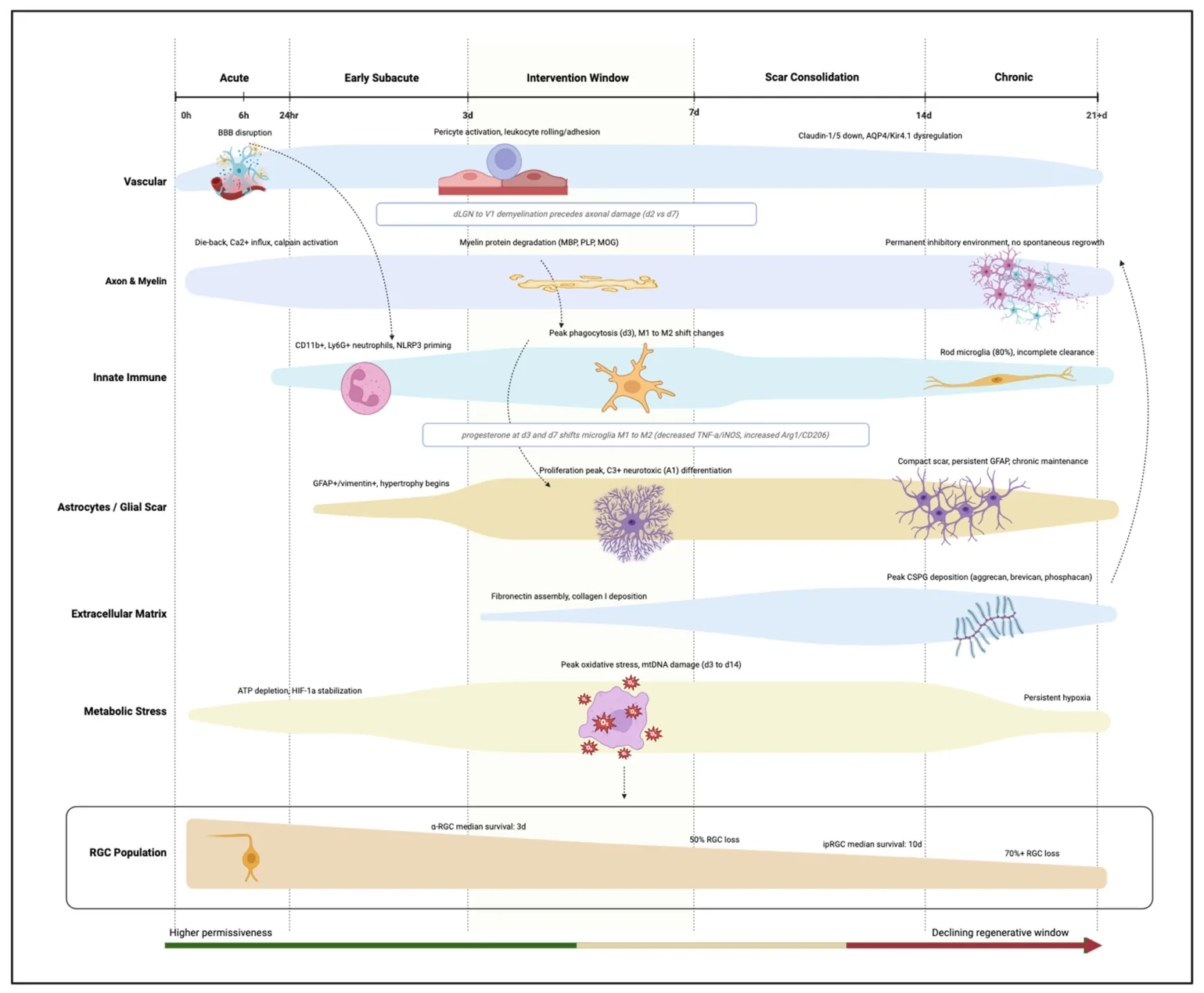
Temporal and interactive model of the extrinsic optic nerve injury microenvironment. Six compartments—vascular, axon and myelin, innate immune, astrocytes and glial scar, extracellular matrix, and metabolic stress—are mapped across the acute (0–24 h), early subacute (1–3 days), intervention window (3–7 days), scar consolidation (7–14 days), and chronic (14–21+days) post-injury phases. Dashed gray arrows represent causal signaling between compartments. The shaded intervention window (days 3–7) marks the period with evidence supporting immunomodulatory efficacy. RGC survival is displayed separately, with median survival for α-RGC and ipRGC subtypes shown alongside overall population decline. The bottom gradient illustrates the decreasing regenerative capacity of the niche over time.

The field of optic nerve regeneration must shift from an exclusive focus on intrinsic regenerative potential to one that also appreciates the critical role of the extrinsic environment. Throughout this paper, we have described the extrinsic environment as a dynamic regenerative niche, emphasizing that these compartments, summarized in Figure 5, coordinate over time rather than function independently. This understanding acknowledges that a multitude of factors contribute to this focus, each of which also exhibits complex and varied interactions with RGCs. However, in synthesis, we can take away broader points that can guide perspective, future research, and therapeutic outlook.

The acute injury environment is conducive to RGC growth. Two major components of the extrinsic environment covered in detail in this review are the extracellular matrix and inflammation. The eye’s status as an immune-privileged site limits infiltration by peripheral immune cells. Compromise of the blood–retinal barrier leads to an unchecked neurotoxic response, in which active immune priming will need to be considered as seen already with methods like zymosan and lens injury, to compensate for limitations posed by immune privilege [228].

Initially, changes that occur post-injury, such as the formation of the glial scar, macrophage recruitment, and microglial debris clearance, play vital roles in promoting regeneration through limiting injury spread and activating pro-regenerative pathways [34,132,136]. Over time, these components shift toward creating a hostile, non-permissive environment for RGC growth. For example, incomplete microglial clearance leaves persistent, pro-inhibitory myelin debris; CSPG accumulation in the glial scar restricts axon extension, and downstream astrocyte activation due to microglial interactions leads to neurotoxicity [20,138,139].

All in all, we propose that extrinsic regulators of growth interact with RGCs in a manner that is temporally dependent and therefore not purely pro- or anti-regenerative. They should thus be targeted as such. For example, restricting glial scar formation leads to failure of regeneration; instead, targeting CSPG formation and interactions in the extracellular matrix is a more viable strategy [34,201,203,204,205]. Promoting acute inflammation via lens injury and zymosan administration is pro-regenerative, whereas inhibiting interactions between inhibitory myelin debris and adverse astrocyte–microglial crosstalk can modulate the negative effects of chronic inflammation [132,220,228,232,244].

While structural challenges, such as ECM remodeling, create large-scale barriers to RGC outgrowth, inhibitory CSPG and myelin constituents impose restrictions on axon growth through molecular interactions [27,39]. Additionally, because the chemical cue gradient in the adult post-injury context differs fundamentally from development, regulators of axon growth, such as Semaphorins and Ephrins, lead to growth inhibition in this altered context [66,67]. However, instead of targeting at an individual scale, targeted therapeutic inhibition should consider their molecular influences on shared pathways, such as the RhoA/ROCK pathway, and its convergence onto growth cone collapse.

### 10.2. Translational Barriers to Combinatorial Therapy

Another fundamental regulator of long-distance regeneration is metabolic capacity; while it is often considered an intrinsically determined factor in RGCs, the influence of the environment on energetic availability should also be emphasized. Membrane order changes observed in sphingolipid and glycerolipid data in the post-injury setting impair mitochondrial capacity, depleting the energy reserve within the axon [159,222]. Therapeutic arms may need to incorporate mitochondrial support, as glycolysis alone is insufficient to sustain transport to the LGN.

In the bigger picture, even when long-distance regeneration is achieved, guidance misdirection, reinnervation, and functional recovery remain vital obstacles to visual restoration [68].

Thus, the components of the extrinsic environment that affect optic nerve regeneration are complex and varied; therefore, potential therapeutics must be correspondingly multifactorial. The environmental complexity discussed is subject to even greater variation when considered alongside patient-specific factors such as age, comorbidities, and disease state.

When this is considered alongside the multitude of intrinsic factors that affect adult CNS regeneration, it becomes clear why combinational, patient-specific therapies are necessary. In optic neuropathies such as glaucoma, regenerative strategies are unlikely to be effective in isolation and should be combined with IOP-lowering protocols as well as vascular and metabolic support to achieve meaningful clinical outcomes.

For example, intrinsic mechanisms influence regenerative outcomes by governing gene expression, metabolism, and cytoskeletal changes within cells, whereas extrinsic cues provide permissive or repulsive signaling in the extracellular environment that influences axonal extension. These mechanisms work in collaboration, as extracellular cues can modify intracellular signaling and vice versa, such that inhibitory extracellular environments can limit regenerative capacity even when intrinsic growth pathways are upregulated [182]. These findings suggest an interplay between intrinsic neuronal growth competence and the extracellular environment, overviewed in Table 4. When assessing axonal injury, it is important to identify which mechanism is impaired to determine whether intrinsic, extrinsic, or combined therapeutic approaches are necessary [245].

### 10.3. Timing, Biomarkers, and Sequencing

The timing of therapeutic intervention is a critical yet underexplored factor in clinical translation. Administering therapy at specific time points may significantly alter the potential for optic nerve regeneration; yet defining this injury window remains a translational challenge. With the inherent variations in injury response when we consider disease chronicity and distinct changes in inflammatory timelines, diseases such as glaucoma require accurate indicators of disease progression.

After injury, the extrinsic environment undergoes substantial changes due to immune and glial responses, shifting from an initially permissive to a chronically inhibitory state. Identifying biomarkers that differentiate acute protective immune responses from chronic neurotoxic conditions is essential. Candidate biomarkers such as CSPG sulfation ratios [20], complement activation [135], astrocyte markers [138], and ceramide: sphingomyelin ratios [155] represent good starting points toward phase-specific therapeutic design. Sequence matters as well: we propose a method that begins with immune priming, proceeds to ECM regulation, then manipulates intrinsic signaling through RhoA/ROCK given its position in downstream convergence and concludes with continued trophic support [10,40,73,222].

### 10.4. Future Methodological Directions

The future of extrinsic modulation should consider integration-focused interventions utilizing emerging technologies such as bioscaffolding, stem cell transplantation, multi-omics, and guidance cue map modification [68,208,209,210,211,212,227,246]. Together, these approaches provide more comprehensive solutions to address the broader environmental and intrinsic mismatch among developmental, pro-regenerative, and adult states.

### 10.5. Patient Selection for Clinical Translation

As research progresses from in vivo and in vitro studies to clinical trials, careful patient selection will be essential for success. Patient-specific factors influence the permissiveness of the extrinsic environment for axon regeneration and determine which populations are most likely to benefit from therapy. For initial trials, early-stage patients with preserved ONH structure, a lack of comorbid vascular abnormalities, intact intraconal anatomy, younger age, evidence of steroid responsiveness, disease chronicity and stage of progression, and reversible visual acuity may represent the most suitable population [247]. The structural integrity of the eye is closely associated with functional outcomes, such as visual field preservation, underscoring the importance of maintaining a healthy ONH [248].

## 11. Conclusions

Achieving functional recovery of the optic nerve requires transforming the environment into one that supports neurite extension, long-distance axonal guidance, and functional reinnervation in the LGN. This understanding highlights that extrinsic components possess both supportive and inhibitory roles, depending on contextual factors such as the individual and injury stage. Consequently, future strategies should focus on optimizing the permissiveness of the extrinsic environment.

In practice, emphasizing the external environment involves moving beyond traditional IOP-lowering methods toward stratified strategies that also consider spatial, metabolic, and temporal barriers to long-distance axon regeneration. We also propose that this will be most effective when used alongside therapies that target intrinsic barriers to growth. Conceptualizing the optic nerve environment as dynamically restorative may reframe regeneration failure and vision loss as modifiable conditions rather than irreversible outcomes.

## Figures and Tables

**Table 1:** Guidance cues.

Cue	Guidance Roles in Optic Pathway Development	Effects on Neurite Outgrowth Post-Injury	General Pathway Influences
**SDF-1/CXCL12**	Through attractive mechanisms, SDF-1 (CXCL12) guides retinal ganglion cell (RGC) axons in the retina to the optic stalk and mediates axon segregation and contralateral tract formation in the optic chiasm [75,76].	Following optic nerve injury, SDF-1 expression is found in glial cells in both the retina and optic nerve, while its receptor, CXCR4, is found in the retinal ganglion cell layer [77]. SDF-1 attractive signaling exerts complex effects. On one hand, SDF-1 stimulates regeneration and attenuates RGC response to inhibitory cues such as myelin and slit-2 [9,51,78]. However, its chemoattraction may prevent distal growth, promoting axonal return towards the injury site instead [79].	SDF-1 (CXC12) activates the G-protein-coupled receptor CXCR4, which has two major downstream effects: PI3K/AKT/mTOR pathway activation and cAMP elevation, which enhances oncomodulin-mediated regeneration [9,51].
**Netrins**	Netrin-1 acts as a chemoattractive cue in the optic nerve head, driving axon growth out of the eye. In distal portions of the optic nerve, Netrin-1 acts as a repellent, confining growing axons within the pathway [80].	In rats, netrin-1 is constitutively expressed by RGCs and glial cells of the optic nerve, but netrin-1 is absent at the optic nerve head both before and after injury. The netrin receptors DCC, UNC5A, and UNC5B have been found to be expressed in RGCs of the uninjured rat optic nerve, but downregulated post-injury, rendering signaling nonfunctional [58,59]. Therefore, the mechanisms through which netrin-1 may impact adult regeneration of RGCs in vivo remain unclear; however, in vitro approaches using RGCs and tissue scaffolding have shown netrin-1 promotes RGC guidance and regeneration [60,61].	In early development, netrin-1 primarily signals through the DCC receptor on RGC growth cones. This activates downstream mediators such as SRC Kinase and the MAP/ERK pathway, promoting axonal growth and attraction [81,82,83]. In later stages of pathway development, Unc5 receptor expression shifts signaling effects towards repulsion via the Rho GTPase pathway [80,84]. Although all three receptors are expressed in the adult optic nerve, post-injury, their expression is downregulated, truncating netrin signaling [58,59].
**MAIs** **(MAG, OMgp,** **Nogo-A)**	Through inhibitory effects, Nogo-A has potential roles in axonal sorting at the optic chiasm and the subsequent facilitation of collateral branching. Greater Nogo-B expression in radial glia located in the midline of the optic chiasm suggests its role in axonal turning at the optic disk and chiasm [85,86].	Myelin debris formed from damaged oligodendrocytes contains myelin-associated inhibitors, whose sustained presence due to incomplete clearance inhibits axonal regeneration [87,88,89].	MAIs signal primarily through the NgR and PirB receptors, leading to RhoA/ROCK pathway activation, and neurite outgrowth inhibition and growth cone collapse [90,91].
**CSPGs**	In the retina, CSPG expression moves peripherally, remaining at the outer edge beyond growing axons forming a boundary for growth through repulsion [92]. In subsequent stages, CSPG regulates axonal divergence at the optic chiasm, sorting of dorsal and ventral axons at the midline, and guidance of axons to superficial regions of the optic tract [93].	Greatly expressed post-injury in the glial scar and acts as a major barrier to axon outgrowth [20]. Greater levels of gliosis and CSPGs are correlated with more severe injury and lower levels of regenerative success [94].	CSPGs exert their inhibitory effects by binding to the PTPσ, LAR, NgR1 and NgR3 receptors [95,96,97]. Downstream, this activates RhoA/ROCK signaling and inactivates Akt and Erk1/2 phosphorylation [98].
**Semaphorins**	Intraretinally, Sema5A and Sema5B contain neurons within the inner plexiform layer (IPL). Sema5A-mediated inhibition has additionally been found to ensheathe RGC axons both at the optic disk and along the length of the optic nerve [99]. At the optic chiasm midline, semaphorins also affect whether axons project contralaterally or ipsilaterally [100,101,102].	Sema3A and Sema5A are found in the retina and by oligodendrocytes respectively post-injury [64,103]. Both are found to inhibit axon growth post-injury [64,66].	Semaphorins lead to growth inhibition through the neuropillin and plexin receptors, which activate the RhoA/ROCK pathway and leads to CRMP phosphorylation through a Cdk5/GSK-3β mediated mechanism [64,66,104,105,106].
**Ephrins**	Within the retina, EphrinB signaling may mediate RGC pathfinding to the optic disk; its inhibitory signaling also mediates the formation of the ipsilateral projection at the optic chiasm [107,108,109]. EphrinA signaling promotes axon repulsion and mediates topographic mapping in the midbrain [110].	Ephrin ligand and receptor expression have been noted in multiple sites post-injury [67,111]. While EphA4 signaling has been noted to be linked to the inhibition of axon outgrowth, studies on EphB2 have found contradictory effects of its activity on the extent of axonal damage [67,111,112].	Ephrin signaling regulates both attractive and repulsive effects dependent on pathway activation. For example, EphA receptors mediate inhibitory effects through both modulation of cyclin-dependent kinase (Cdk) 5 activity and RhoA activation with Cdc42 and Rac1 inhibition [67,113,114]. EphB receptors mediate repulsive signaling through RhoA activation in both forward and reverse signaling [70,115]. Rac1 activation through the guanine nucleotide-exchanging factor, Tiam1, mediates neurite outgrowth through Ephrin-B1 reverse signaling and EphA2 forward signaling [116].
**Slit proteins**	Slit proteins restrict RGC growth towards the optic disk to the retinal optic fiber layer (OFL) [117]. Through further inhibitory signaling, they have roles in the sorting of axons at the optic chiasm and the relative position of the structure on the midline [118,119]	Slit1 expression is absent in the uninjured optic nerve but is transiently expressed days after injury [41]. Slit/Robo repulsive signaling leads to the failure of axons to innervate targets such as neurons in the suprachiasmatic nucleus (SCN) [120].	Slit proteins act as repulsive guidance cues through binding Robo receptors, whose intracellular domain recruits Slit-Robo GTPase-activating proteins (srGAPs) that inactivate Cdc42, a stimulator of neurite outgrowth [121].
**Laminin**	Laminin signaling orients the emergence of RGC axons [122]. Both prior to and during RGC outgrowth, laminin acts as a growth-permissive substrate throughout the optic nerve pathway, delineating the correct path for neuronal outgrowth [123,124]	Expression of laminin both prior and post optic nerve injury in adult organisms is not found in low regenerative organisms [125,126]. However, exogenous laminin administration in these organisms promotes axonal growth in the optic nerve [127].	Laminin-Integrin interactions activate focal adhesion kinase (FAK); subsequent FAK/Src signaling activates pro-regenerative pathways including PI3K/Akt and MEK/MAPK [128,129,130,131].

**Table 2:** Temporal considerations in optic nerve injury.

Component	Early Protective/Developmental Role	Post-Injury Inhibitory Role
**Glial Scar**	Forms a protective barrier after injury, preventing surrounding tissue from secondary damage [34,148].	Accumulated CSPGs act as a physical barrier to axonal regeneration [20].
**Guidance cues**	During development, directs axon pathfinding to correct targets [99].	Post-injury, repulsive molecules such as semaphorin and MAIs prevent axonal growth and promote growth cone collapse [149,150]
**Debris**	Triggers inflammatory responses required for clearance and healing [151].	Residual debris that result from incomplete clearance may lead to neuroinflammation and secondary tissue damage [89].
**Inflammation**	Acute activation may contribute to debris clearance and provide neurotrophic support [140].	Chronic immune activation can exacerbate tissue damage, suppress oligodendrocyte precursor cell differentiation and lead to RGC death [134].

**Table 4:** Overview of barriers, mediators, signaling, and interventions.

Extrinsic Barrier	Major Molecular Mediators	Downstream Signaling	Interventions
Extracellular matrix remodeling/glial scar	CSPGs (aggrecan, versican, neurocan, brevican), tenascin-C, CHST-11	PTPσ/LAR/NgR1/NgR3, RhoA/ROCK; inhibition of Akt/ERK; Piezo ion channels	ChABC, ARSB, CHST-11 inhibition, hyaluronidase, ECM bioscaffolds, synthetic scaffolds
Myelin-associated inhibition	Nogo-A, MAG, OMgp	NgR1/PirB, RhoA/ROCK	NgR1(310)-Fc, ROCK inhibitors, C3 transferase
Repulsive guidance cues	Semaphorins, ephrins, Slit proteins	Plexins/neuropilins, Eph receptors, Robo receptors RhoA/ROCK, CRMP2, Cdc42/Rac1	CRMP2 modulation, EphA4 inhibition
Loss of permissive signaling	Reduced laminin, impaired NTF signaling, reduced netrin receptor expression	Integrin/FAK PI3K/Akt, MAPK/ERK; CXCR4, PI3K/Akt/mTOR; CNTF, JAK/STAT	Laminin support, CNTF, BDNF, GDNF, SDF1/CXCL12, mesenchymal stem cells
Neuroinflammation	Activated microglia/macrophages, complement, TNF-α, IL-1α, astrocytes	TLR2/Dectin-1, GLP-1 signaling, complement pathways	Lens injury, zymosan, Pam3Cys, β-glucan, GLP-1 agonists, modulation of microglia–astrocyte
Metabolic and vascular constraints	Mitochondrial dysfunction, ceramides, sphingomyelin, impaired blood flow	Reduced ATP production, altered lipid signaling, oxidative stress	Mitochondrial/metabolic support, lipid-targeted therapies

## Data Availability

No new data were created or analyzed in this study. Data sharing is not applicable to this article.

## Abbreviations

The following abbreviations are used in this manuscript:

ACG	Acute angle-closure glaucoma
APC	Article processing charge
ARSB	Arylsulfatase B
ATP	Adenosine triphosphate
BDNF	Brain-derived neurotrophic factor
cAMP	Cyclic adenosine monophosphate
CCL2	C-C motif chemokine ligand 2
ChABC	Chondroitinase ABC
CHST-11	Carbohydrate sulfotransferase-11
CNS	Central nervous system
CNTF	Ciliary neurotrophic factor
Col1α1	Collagen type 1 alpha 1
CRMP2	Collapsin response mediator protein 2
CS-A	4-sulfated chondroitin sulfate
CSPGs	Chondroitin sulfate proteoglycans
CXCL5	C-X-C motif chemokine ligand 5
CXCR4	C-X-C motif chemokine receptor 4
DCC	Deleted in colorectal cancer
DM	Diabetes mellitus
ECM	Extracellular matrix
EphR	Ephrin receptor
FAK	Focal adhesion kinase
GAG	Glycosaminoglycan
GDNF	Glial cell line-derived neurotrophic factor
GLP-1	Glucagon-like peptide-1
ILP/ISP	Intracellular sigma peptide/intracellular LAR peptide
IOP	Intraocular pressure
JAK-STAT	Janus kinase/signal transducer and activator of transcription
LAR	Leukocyte common antigen-related
LGN	Lateral geniculate nucleus
LIMK	LIM domain kinase
MAGs	Myelin-associated glycoproteins
MAIs	Myelin-associated inhibitors
MAPK/ERK	Mitogen-activated protein kinase/extracellular signal-regulated kinase
MLC	Myosin light chain
mTOR	Mechanistic target of rapamycin
NAION	Non-arteritic anterior ischemic optic neuropathy
NgR	Nogo receptor
NTF	Neurotrophic factor
NTG	Normal tension glaucoma
Ocm	Oncomodulin
OMgp	Oligodendrocyte myelin glycoprotein
ONC	Optic nerve crush
ONH	Optic nerve head
OPP	Ocular perfusion pressure
OSA	Obstructive sleep apnea
PI3K/Akt	Phosphoinositide 3-kinase/protein kinase B
POAG	Primary open-angle glaucoma
PTEN	Phosphatase and tensin homolog
PTPσ	Protein tyrosine phosphatase sigma
RGC	Retinal ganglion cell
RhoA/ROCK	Ras homolog family member A/Rho-associated coiled-coil containing protein kinase
ROS	Reactive oxygen species
SDF1/CXCL12	Stromal cell-derived factor 1
TLR-2	Toll-like receptor 2
TON	Traumatic optic neuropathy
